# Impact of sunlight exposure on the age of onset of bipolar disorder

**DOI:** 10.1192/j.eurpsy.2022.1055

**Published:** 2022-09-01

**Authors:** N. Arfaoui, J. Hamdoun, D. Bougacha, H. Ben Ammar, Z. El Hechmi

**Affiliations:** 1 Razi Hospital, Department Of Psychiatry Aziza Othmana, Mannouba, Tunisia; 2 Razi hospital, F, TUNIS, Tunisia; 3 Razi hospital, Psychiatry F, manouba, Tunisia

**Keywords:** bipolar disorder, age, sunlight exposure, onset

## Abstract

**Introduction:**

Bipolar disorder is a multifactorial disorder influenced by multiple genetic and environmental factors.There is limited understanding of how non-genetic factors may impact the age of onset of bipolar disorder

**Objectives:**

To study the age of onset of bipolar disease in Tunisia (where the average duration of sunshine is 8 hours/day) and compare it to the age of onset in countries with a lower duration of sunshine (Germany 0.17h/day; Norway 1.40h/day).

**Methods:**

We conducted a retrospective study of 100 patients with bipolar disorder type I followed at the psychiatric department Aziza Othmana at Razi hospital.The data collection was done using a pre-established paper form exploring sociodemographic and clinical data.The duration of sunshine was estimated according to the average number of hours of sunshine per day in each country collected through meteorological sites.

**Results:**

Our population was predominantly male (60%) with a mean age of 48.7 years.The first episode was manic in 76% of cases. The mean age of onset in our sample was 25.86 years, with extremes ranging from 13 to 49 years.An early onset (threshold age=21 years) was found in 36% of the Tunisian population. The age of onset was earlier in patients with a family history of bipolar disorder: 22.76 years vs 28.23 years. A late onset (threshold age=37 years) was found in 13% of the Tunisian population.

**Conclusions:**

The study confirmed that there is an inverse relationship between the degree of sunlight and the age of onset of the disease, especially in the presence of a family history of mood disorders

**Disclosure:**

No significant relationships.

